# Race is a risk factor for calcinosis in patients with JDM – early results from the CARRAnet registry study

**DOI:** 10.1186/1546-0096-10-S1-A65

**Published:** 2012-07-13

**Authors:** Mark F Hoeltzel, Mara L Becker, Angela B Robinson, Brian M Feldman, Adam Huber, Ann M Reed

**Affiliations:** 1 Children's Mercy Hospital, Kansas City, MO, USA; 2 IWK Health Centre, Halifax, NS, Canada; 3 Mayo Clinic, Rochester, MN, USA; 4 Rainbow Babies and Childrens Hospital, Cleveland, OH, USA; 5 The Hospital for Sick Children, Toronto, ON, Canada; 6 Stanford, CA, USA

## Purpose

Previously established risk factors for calcinosis include duration of disease and the length of time to treatment, suggesting early aggressive treatment may be important in preventing calcinosis. Race has also been suggested to be a risk factor, but this observation was thought to be secondary to delays in treatment. We utilized the CARRAnet registry to investigate potential risk factors for calcinosis.

## Methods

Children with juvenile dermatomyositis (JDM) in the Childhood Arthritis and Rheumatology Research Alliance (CARRA) registry study were included in this analysis. Race and ethnicity were self-reported. Subjects with current or past calcinosis were identified, and bi-variable associations between clinical variables and calcinosis were evaluated by Chi-square test, Fisher exact test, two-sample t-test, or Wilcoxon Rank Sum test as appropriate. A multivariable stepwise logistic regression model was run that included variables that were significantly associated in the bi-variable analysis, defined by *P*<0.1. All statistical analyses were conducted using JMP Stats version 8.0 (SAS Institute, Cary, NC).

## Results

Prior to December 28, 2010, 102 subjects meeting modified Bohan and Peter criteria for JDM were enrolled from 23 U.S. sites. Fifteen subjects were excluded due to lack of data regarding calcinosis or race. Ten of the remaining 87 patients had a history of calcinosis. Potential predictors of calcinosis identified *a priori* included race, ethnicity, sex, ANA status, age, age of onset, duration of disease, and time to rheumatologic care. Bi-variable analyses revealed a significant association between calcinosis and African American (AA) race (*P*=0.029), disease duration (*P*=0.005), absence of antinuclear antibodies (*P*=0.024), and male gender (*P*=0.037) (Table 1). Analyzing these four variables in a multiple stepwise logistic regression model revealed that AA ancestry was the most significant predictor of calcinosis (OR [95% CI] 5.8 [1.3, 24.8] *P*=0.019). When demographic variables and outcome assessments were compared between AA patients and non-AA patients, only CHAQ scores were statistically significantly different in AA patients (mean (±SD), 0.54 (±0.51) vs. 0.29 (±0.52) in non-AA patients [*P*= 0.033]).

**Table 1:** Analysis of calcinosis risk factors

	Number (%) or Mean (+/-SD) *with* calcinosis	Bi-variable analysis (p-value)	Stepwise logistic regression analysis (OR [95%CI] *p* value
African American ancestry	4 (33%)	0.03*	5.8 [1.3, 24.8] 0.02*
Non-AA ancestry	6 (8%)		
Male	5 (26%)	0.04*	NS
Female	5 (7%)		
Negative ANA	6 (19%)	0.02*	NS
Positive ANA	1 (2%)		
Disease duration, yrs	7.2 (+/-4.1)	<0.01*	NS

## Conclusion

Children with JDM of African American ancestry have increased odds of developing calcinosis, even after controlling for duration of disease and time to rheumatologic care. Male gender, negative ANA status, and disease duration were associated with calcinosis in bi-variable analysis, however, these variables were limited by incomplete data capture, which may have affected the results of the multi-variable regression model. Analysis of a larger more complete cohort is needed to confirm these observations.

## Disclosure

Mark F. Hoeltzel: None; Mara L. Becker: None; Angela B. Robinson: None; Brian M. Feldman: None; Adam Huber: None; Ann M. Reed: None; Juvenile Myositis CARRA Subgroup: None; CARRAnet Investigators: None.

